# Congenital Syphilis Prevention Challenges, Pacific Coast of Colombia, 2018–2022

**DOI:** 10.3201/eid3005.231273

**Published:** 2024-05

**Authors:** Jose F. Fuertes-Bucheli, Diana P. Buenaventura-Alegría, Adriana M. Rivas-Mina, Robinson Pacheco-López

**Affiliations:** Universidad Icesi, Cali, Colombia (J.F. Fuertes-Bucheli, R. Pacheco-López);; Centro Internacional de Entrenamiento e Investigaciones Médicas–CIDEIM, Cali (J.F. Fuertes-Bucheli);; Hospital Distrital Luis Ablanque de la Plata, Buenaventura, Colombia (D.P. Buenaventura-Alegría);; Universidad Libre, Cali (D.P. Buenaventura-Alegría, A.M. Rivas-Mina)

**Keywords:** congenital syphilis, pregnancy complications, infectious, pregnancy tests, mass screening, early diagnosis, social vulnerability, health disparate, minority and vulnerable populations, Colombia, bacteria

## Abstract

High incidences of congenital syphilis have been reported in areas along the Pacific coast of Colombia. In this retrospective study, conducted during 2018–2022 at a public hospital in Buenaventura, Colombia, we analyzed data from 3,378 pregnant women. The opportunity to prevent congenital syphilis was missed in 53.1% of mothers because of the lack of syphilis screening. Characteristics of higher maternal social vulnerability and late access to prenatal care decreased the probability of having >1 syphilis screening test, thereby increasing the probability of having newborns with congenital syphilis. In addition, the opportunity to prevent congenital syphilis was missed in 41.5% of patients with syphilis because of the lack of treatment, which also increased the probability of having newborns with congenital syphilis. We demonstrate the urgent need to improve screening and treatment capabilities for maternal syphilis, particularly among pregnant women who are more socially vulnerable.

Congenital syphilis is an infectious disease that is transmitted from a mother with syphilis to the fetus during pregnancy or childbirth and is caused by the bacterium *Treponema pallidum* (*1*). Globally, congenital syphilis is an infectious disease of high interest for public health but is occasionally neglected and requires collaborative actions for its control (*2*,*3*). In the Americas, congenital syphilis incidence has increased from 0.38/1,000 live births in 2009 to 0.61/1,000 live births in 2020 (*4*). This concerning trend underscores the importance of addressing this disease, which constitutes a major global cause of fetal loss, stillbirths, neonatal death, and congenital infection (*1*,*5*,*6*).

Comprehensive interventions involving diverse stakeholders in the healthcare system and community are crucial to preventing and controlling maternal and congenital syphilis, and those align with the third of the Sustainable Development Goals adopted by the United Nations Member States (*2*,*7*). Strategies used in local programs and shared globally include promoting condom use, ensuring timely access to antenatal care, early gestational syphilis detection through prompt point-of-care screening, and treating infections in a timely manner (*8*). Education on sexual and reproductive health, along with implementing epidemiologic surveillance, are also part of those efforts to prevent and control maternal and congenital syphilis (*9*). However, identifying and prioritizing populations for the specific reinforcement of those strategies in low- to middle-resource contexts is imperative.

In Colombia, a substantial increase in maternal syphilis prevalence was observed during 2017–2021; prevalence rose from 7.8 to 16.2 cases/1,000 newborns (live births and stillbirths). In 2021, maternal syphilis prevalence in Buenaventura alone was 45.8 cases/1,000 newborns, and congenital syphilis incidence also exceeded the national incidence (7.2 vs. 3.2 cases/1,000 newborns) (*10*), leading to the reporting of an epidemic in that area (*11*). Previous studies have aimed to assess the reason for this; a study conducted by Cruz et al. (*11*) in the same area found that only 8% of pregnant women received adequate treatment. Another study in South America revealed that congenital syphilis incidence is elevated in newborns of young Afro-American women who have lower educational attainment and women lacking prenatal care (*12*), elements that could be indicative of social vulnerability (*13*,*14*).

We sought to identify the characteristics (sociodemographic factors, obstetric history, and level of syphilis screening and treatment) of pregnant women enrolled in a prenatal care program (PCP) on the Pacific coast of Colombia associated with having newborns with congenital syphilis and the lack of maternal syphilis screening. In addition, we explored the characteristics of mothers with syphilis associated with having newborns with congenital syphilis.

## Methods

### Design

We conducted an analytical retrospective cohort study during January 2018–December 2022 in Buenaventura, Colombia. We used records of pregnant women enrolled in a PCP of a public hospital. 

### Study Area

Buenaventura is the main city on the Pacific coast of Colombia. The most recent population census in 2018 reported 258,445 inhabitants in the city, of whom 86.7% identify as Afro-Colombian (*15*). In contrast, at the national level, Afro-Colombians make up only 9.34% of Colombia's total population and are predominantly concentrated along the Pacific and Caribbean coasts of the country (*16*). Afro-Colombians face elevated levels of multidimensional poverty, marked by disparities in occupation type, educational attainment, school dropout rates, literacy, and access to healthcare services (*16*).

### Study Population

We included records of pregnant women of all ages who accessed a PCP in the main referral public hospital in Buenaventura (*11*) and whose babies were born in that hospital, with or without congenital syphilis. We excluded records of persons with multiple pregnancies and records with duplicate or incomplete data.

### Variables and Definitions

Sociodemographic variables were age; ethnicity (Afro-Colombian or others); rural or urban area of residence; educational level, basic or lower (completed secondary school or lower) or postsecondary (technical, university, or higher); socioeconomic level, on the basis of a 1–6 scale (low-low, low, low-middle, middle, middle-high, and high) approximating the hierarchical socioeconomic difference from poverty to wealth in Colombia (*17*); marital status: single or with partner (de facto marriage or married); position as head of household (a person, whether single or married, who bears responsibility for providing financial or social support to their dependents); and the type of health coverage (*18*). We regarded the lowest categories within the outlined sociodemographic factors as characteristics of higher maternal social vulnerability factors. Other variables consisted of past pregnancy history (number of pregnancies, spontaneous abortions, and stillbirths); the gestational trimester in which prenatal care access was obtained; and whether syphilis screening had been performed and, if it was diagnosed, whether there had been a lack of treatment, defined as the lack of >1 dose of benzathine penicillin G (BPG) >30 days before delivery (*9*,*19*).

We applied the operational definitions of maternal and congenital syphilis according to the clinical practice guide issued by the Ministry of Health and Social Protection of Colombia (*9*). A patient with detected maternal syphilis was any mother with a diagnosis of syphilis during prenatal care, with or without clinical signs, who had a positive rapid point-of-care treponemal test accompanied by a reactive nontreponemal test at any dilution and who had not received adequate treatment or had an untreated reinfection (*9*).

A newborn with congenital syphilis was any live birth or stillbirth that met >1 of the following criteria: newborns of a mother with untreated syphilis or inadequate treatment (without >1 dose of BPG >30 days before delivery) to prevent congenital syphilis (*9*,*19*,*20*), regardless of the result of the nontreponemal test of the newborn; any newborn with nontreponemal test titers 4 times higher than the mother’s titers at the time of delivery, which is equivalent to 2 dilutions above the maternal titer; any newborn of a pregnant person whose syphilis was diagnosed during that pregnancy and with >1 clinical manifestations suggestive of congenital syphilis on physical examination, along with paraclinical tests suggestive of the infection; or any newborn with demonstrated *T. pallidum* in laboratory tests (*9*).

### Data Sources

The hospital PCP provided the database, which included sociodemographic information, gestational age at the start of prenatal care, and obstetric history, as well as screening and treatment data for syphilis. Moreover, the database indicated whether the newborn was classified as a congenital syphilis case-patient. However, the database lacked details on the clinical stage of maternal syphilis and further details of the newborn, and although it reported the reactive or nonreactive result of the nontreponemal test for syphilis, it did not include the result in dilutions.

### Statistical Analysis

We organized the data in Excel 365 (Microsoft, https://www.microsoft.com) and conducted analyses using Stata 14.0 (StataCorp LLC, https://www.stata.com). We conducted an exploratory analysis of the database to identify outliers or missing data. We reported categorical variables as frequencies and percentages and continuous variables as medians and interquartile ranges (IQRs). We determined the annual percentage of congenital syphilis cases by dividing the number of newborns with the infection by the total number of newborns for each year and multiplying by 100.

To identify maternal factors associated with having newborns with congenital syphilis, we compared pregnant women whose newborns had congenital syphilis with those whose newborns did not. Furthermore, to assess factors associated with the lack of maternal syphilis screening, we compared pregnant women who had >1 rapid point-of-care treponemal test through the PCP with those who did not. For those 2 objectives, we used 2 × 2 tables and calculated crude odds ratios with their respective 95% CIs. We assessed statistical significance using χ^2^ and Mann-Whitney U tests as appropriate. We conducted multivariable analyses through multiple logistic regression, and each initial or saturated model included variables with p<0.25 in the bivariate analysis, following the approach of Hosmer et al. (*21*), along with other variables considered to reflect social vulnerabilities. We selected the most parsimonious model by using the likelihood ratio test.

We conducted a subanalysis to identify factors in mothers with syphilis associated with having newborns with congenital syphilis. We compared patients with detected maternal syphilis during PCP and who had newborns with congenital syphilis to those who did not. We assessed the probability of having newborns with congenital syphilis using the relative risk (RR) as a measure of association. We determined RRs and corresponding 95% CIs through bivariate analysis. This research was conducted following the Declaration of Helsinki and was approved by the Human Research Ethics Committee of the Universidad Libre under protocol #010.

## Results

### General Description

During the study period, 5,172 admissions of pregnant women to the PCP were identified. Among those, we excluded 1,589 (30.7%) duplicate records and 205 (4.0%) records with insufficient information. In total, we analyzed 3,378 records. The median age was 24 (IQR 20–29) years, 98.5% were Afro-Colombian, 95.6% were from urban areas, 94.9% were at a basic or lower educational level, 93.8% were at the lowest socioeconomic level, 100% had subsidized health insurance, 78.7% were heads of households, and 19.1% had single marital status.

The median gestational age at initiation of the PCP was 12.3 (IQR 8.6–18.7) weeks, but 53.3% accessed the PCP in the first trimester of pregnancy. In total, 63.3% (2,139) women had >1 syphilis screening test; 270 had a positive rapid point-of-care treponemal test. Of those 270 patients, 86.7% (234) were considered cases with reactive nontreponemal tests, 11.1% (30) did not undergo the nontreponemal test, and 2.2% (6) had a nonreactive nontreponemal test. Finally, 96 mothers with newborns with congenital syphilis were reported.

**Figure 1:**
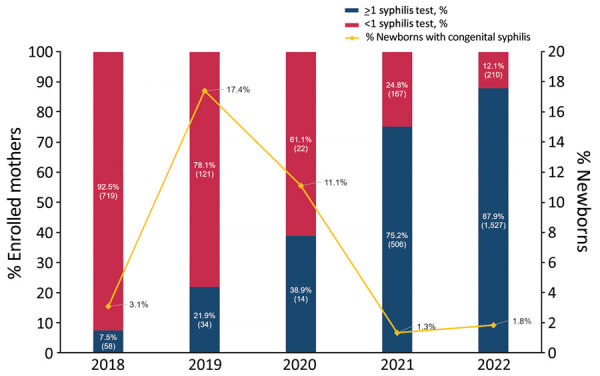
Syphilis screening and percentage of newborns with congenital syphilis by year of mothers’ entry into the prenatal care program at a public hospital, Buenaventura, Colombia, 2018–2022. Maternal syphilis screening has improved progressively over the years, though the number of mothers who participated in the prenatal care program dropped in 2020. In addition, variability was observed in the percentage of newborns with congenital syphilis, which decreased from 3.1% to 1.8% in the evaluated period, although the percentage increased slightly from 2021 to 2022. Scales for the y-axes differ substantially to underscore patterns but do not permit direct comparisons.

The percentage of pregnant women screened for syphilis improved over time, increasing from <10% in 2018 to nearly 90% in 2022 (Figure 1). The number of participants entering the PCP each year varied; the lowest number was in 2020 and the highest was in 2022. The percentage of newborns with congenital syphilis per year decreased from 3.1% in 2018 to 1.8% in 2022 but increased during the interim years, 2019 and 2020 (Figure 1).

### Factors Associated with Having Newborns with Congenital Syphilis

Of all pregnant women enrolled in the PCP, 53.1% who had newborns with congenital syphilis were not screened in the PCP. In the bivariate analysis, not having been screened through the PCP was associated with the probability of having newborns with congenital syphilis (adjusted odds ratio [aOR] 1.99, 95% CI 1.32–3.00; p = 0.001) (Table 1).

**Table 1:** Bivariate analysis of factors associated with having newborns with congenital syphilis in mothers who entered in a prenatal care program of a public hospital in Buenaventura, Colombia, 2018–2022*

Characteristic	Newborn with congenital syphilis		Newborn without congenital syphilis		Crude OR (95% CI)	p value
Age group, y						
<18						
Yes	19 (19.8)		592 (18.0)		1.12 (0.63–1.89)	0.659
No	77 (80.2)		2,690 (82.0)		Referent	
19–34						
Yes	72 (75.0)		2,403 (73.2)		1.09 (0.68–1.83)	0.697
No	24 (25.0)		879 (26.8)		Referent	
>35						
Yes	5 (5.2)		287 (8.7)		0.57 (0.18–1.40)	0.224†
No	91 (94.8)		2,995 (91.3)		Referent	
Educational level						
Basic or lower‡	92 (95.8)		3,115 (94.9)		1.23 (0.45–4.67)	0.684
Postsecondary	4 (4.2)		167 (5.1)		Referent	
Socioeconomic stratum§						
1 (low-low)	94 (97.9)		3,075 (93.7)		3.16 (0.84–26.68)	0.090†
2 (low)	2 (2.1)		207 (6.3)		Referent	
Residential area						
Rural	1 (1.0)		148 (4.5)		0.22 (0.01–1.29)	0.102†
Urban	95 (99.0)		3,134 (95.5)		Referent	
Ethnicity						
Afro-Colombian	92 (95.8)		3,234 (98.5)		0.34 (0.12–1.33)	0.033†
Other	4 (4.2)		48 (1.5)		Referent	
Occupation						
Head of household¶	77 (80.2)		2,580 (78.6)		1.1 (0.65–1.94)	0.706
Other	19 (19.8)		702 (21.4)		Referent	
Marital status						
Single	24 (25.0)		621 (18.9)		1.42 (0.85–2.31)	0.135†
With partner#	72 (75.0)		2,661 (81.1)		Referent	
History of >3 pregnancies						
Yes	33 (34.4)		1,024 (31.2)		1.15 (0.73–1.80)	0.508
No	63 (65.6)		2,258 (68.8)		Referent	
History of spontaneous abortions						
Yes	23 (24.0)		655 (20.0)		1.26 (0.75–2.1)	0.334
No	73 (76.0)		2,627 (80.0)		Referent	
History of stillbirths						
Yes	3 (3.1)		89 (2.7)		1.16 (0.23–3.60)	0.806
No	93 (96.9)		3,193 (97.3)		Referent	
Trimester of access to PCP						
Second or third	50 (52.1)		1,526 (46.5)		1.25 (0.81–1.92)	0.279
First	46 (47.9)		1,756 (53.5)		Referent	
>1 syphilis screening test						
No	51 (53.1)		1,188 (36.2)		1.99 (1.30–3.07)	<0.001†
Yes	45 (46.9)		2,094 (63.8)		Referent	

*Values are no. (%) except as indicated. PCP, prenatal care program. †Indicates variables with a p value of **<**0.25 included in the logistic regression. ‡Basic or lower: completed secondary school or lower; post-secondary: technical, technologist, university, or higher. §Socioeconomic stratum scale in Colombia: 1 to 6, with 1 being the lowest level. ¶Person, whether single or married, who bears the ongoing responsibility for providing financial or social support to their minor newborns or other dependents. #De facto marriage or married.

### Factors Associated with the Lack of Syphilis Screening

In the bivariate analysis, age of <18 years or >35 years, basic or lower education, low socioeconomic status, single marital status, and accessing the PCP in the second or third trimester of gestation were associated with a lack of syphilis screening (Table 2). On the other hand, Afro-Colombian ethnicity and obstetric history were identified as protective factors.

**Table 2:** Factors associated with having >1 screening test for syphilis in a prenatal care program of a public hospital, Buenaventura, Colombia, 2018–2022*

Characteristic	>1 syphilis screening test				Crude OR (95% CI)	p value	
	No		Yes				
Age group, y							
**<**18							
Yes	258 (20.8)		353 (16.5)		1.33 (1.11–1.60)	0.001†	
No	981 (79.2)		1,786 (83.5)		Referent		
19–34							
Yes	897 (72.4)		1,578 (73.8)		0.93 (0.79–1.09)	0.383	
No	342 (27.6)		561 (26.2)		Referent		
**>**35							
Yes	84 (6.8)		208 (9.7)		0.67 (0.51–0.88)	0.003†	
No	1,155 (93.2)		1,931 (90.3)		Referent		
Educational level‡							
Basic or lower	1,198 (96.7)		2,009 (93.9)		1.89 (1.31–2.77)	<0.001†
Postsecondary	41 (3.3)		130 (6.1)		Referent	
Socioeconomical stratum§							
1: low-low	1,205 (97.3)		1,964 (91.8)		3.16 (2.16–4.74)	<0.001†
2: low	34 (2.7)		175 (8.2)		Referent	
Residential area							
Rural	52 (4.2)		97 (4.5)		0.92 (0.64–1.31)	0.644
Urban	1,187 (95.8)		2,042 (95.5)		Referent	
Ethnicity							
Afro-Colombian	1,197 (96.6)		2,129 (99.5)		0.13 (0.06–0.27)	<0.001†
Other	42 (3.4)		10 (0.5)		Referent	
Occupation							
Head of household¶	987 (79.7)		1,670 (78.1)		1.09 (0.92–1.31)	0.278#
Other	252 (20.3)		469 (21.9)		Referent	
Marital status							
Single	326 (26.3)		319 (14.9)		2.04 (1.71–2.43)	<0.001†
With partner**	913 (73.7)		1,820 (85.1)		Referent	
History of ≥3 pregnancies							
Yes	321 (25.9)		736 (34.4)		0.67 (0.57–0.78)	<0.001†	
No	918 (74.1)		1,403 (65.6)		Referent		
History of spontaneous abortions							
Yes	201 (16.2)		477 (22.3)		0.67 (0.56–0.81)	<0.001†	
No	1,038 (83.8)		1,662 (77.7)		Referent		
History of stillbirths							
Yes	23 (1.9)		69 (3.2)		0.57 (0.34–0.93)	0.018†
No	1,216 (98.1)		2,070 (96.8)		Referent	
Trimester of access to PCP							
Second or third	625 (50.4)		951 (44.5)		1.27 (1.10–1.47)	<0.001†
First	614 (49.6)		1,188 (55.5)		Referent	

*Values are no. (%) except as indicated. PCP, prenatal care program. †Indicates variables with a p value of **<**0.25 included in the logistic regression. ‡Basic or lower: completed secondary school or lower; postsecondary: technical, university, or higher. §Socioeconomic stratum scale in Colombia: 1 to 6, with 1 being the lowest level. ¶Person, whether single or married, who bears the ongoing responsibility for providing financial or social support to their dependents. #Was included in the multivariate analysis for the importance in Colombian social context. **De facto marriage or married.

**Figure 2:**
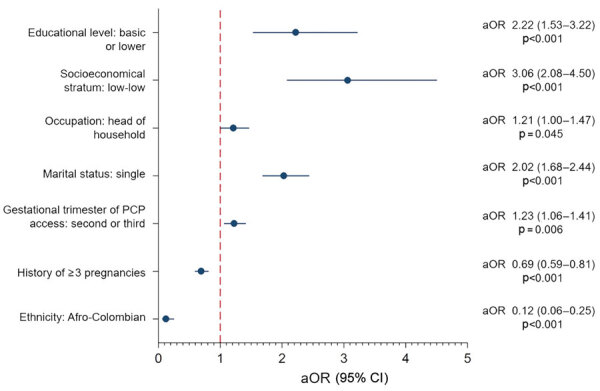
Multivariate analysis of factors associated with the lack of >1 maternal syphilis screening test in prenatal care program, Buenaventura, Colombia, 2018–2022. Vertical red dashed line indicates the nonassociation reference point. aOR, adjusted odds ratio; PCP, prenatal care program.

Multivariate analysis revealed that independent factors associated with the lack of screening during the PCP were basic or lower educational level (aOR 2.22), lowest socioeconomic status (aOR 3.06), occupation as head of household (aOR 1.21), single marital status (aOR 2.02), and accessing the PCP in the second or third trimester of pregnancy (aOR 1.23) (Figure 2). Conversely, having had >3 previous pregnancies (aOR 0.69) and being of Afro-Colombian ethnicity (aOR 0.12) were identified as protective factors.

### Factors of Mothers with Syphilis Associated with Having Newborns with Congenital Syphilis

In 234 pregnant patients, syphilis was detected during prenatal care; 41 had newborns with congenital syphilis. Of those 41 patients, 41.5% did not receive >1 dose of BPG >30 days before delivery, which was associated with a 4.31-fold increase in the probability of having newborns with the infection (Table 3).

**Table 3:** Analysis of maternal factors associated with having a newborn with congenital syphilis in pregnant women with syphilis at a public hospital, Buenaventura, Colombia, 2018–2022*

Characteristic	Newborn with congenital syphilis		Newborn without congenital syphilis		RR (95% CI)	p value	
Age group, y							
<18							
Yes	9 (22.0)		35 (18.1)		1.21 (0.62–2.35)	0.570	
No	32 (78.0)		158 (81.9)		Referent		
19–34							
Yes	30 (73.2)		146 (75.6)		0.89 (0.48–1.67)	0.738	
No	11 (26.8)		47 (24.4)		Referent		
>35 y							
Yes	2 (4.9)		12 (6.2)		0.80 (0.21–2.99)	0.742	
No	39 (95.1)		181 (93.8)		Referent		
Educational level†							
Basic or lower	40 (97.6)		185 (95.9)		1.60 (0.24–10.37)	0.605
Postsecondary	1 (2.4)		8 (4.1)		Referent	
Socioeconomical stratum‡							
1: low-low	41 (100)		184 (95.3)		NA (NA)	0.158
2: low	0		9 (4.7)		Referent	
Residential area							
Rural	0		7 (3.6)		NA (NA)	0.215
Urban	41 (100)		186 (96.4)		Referent	
Ethnicity							
Afro-Colombian	41 (100)		193 (100)		NA (NA)	NA
Other	0		0		Referent	
Occupation							
Head of household§	33 (80.5)		155 (80.3)		1.00 (0.50–2.03)	0.979
Other	8 (19.5)		38 (19.7)		Referent	
Marital status							
Single	11 (26.8)		46 (23.8)		1.13 (0.61–2.12)	0.684
With partner¶	30 (73.2)		147 (76.2)		Referent	
History of >3 pregnancies							
Yes	11 (26.8)		76 (39.4)		0.61 (0.32–1.17)	0.131	
No	30 (73.2)		117 (60.6)		Referent		
History of spontaneous abortions							
Yes	9 (22.0)		40 (20.7)		1.06 (0.54–2.07)	0.860	
No	32 (78.0)		153 (79.3)		Referent		
History of stillbirths							
Yes	1 (2.4)		8 (4.1)		0.62 (0.09–4.05)	0.605
No	40 (97.6)		185 (95.9)		Referent	
Trimester of access to PCP							
Second or third	22 (53.7)		87 (45.1)		1.32 (0.76–2.31)	0.317
First	19 (46.3)		106 (54.9)		Referent	
Lack of syphilis treatment#							
Yes	17 (41.5)		16 (8.3)		4.31 (2.62–7.12)	<0.001
No	24 (58.5)		177 (91.7)		Referent	

*Values are no. (%) except as indicated. BPG, benzathine penicillin G; PCP, prenatal care program; RR, relative risk. †Basic or lower: completed secondary school or lower; postsecondary: technical, university, or higher. ‡Socioeconomic stratum scale in Colombia: 1 to 6, with 1 being the lowest level. §Person, whether single or married, who bears the ongoing responsibility for providing financial or social support to their dependents. ¶De facto marriage or married. #Lack of >1 dose of BPG >30 d before delivery.

## Discussion

In this study conducted in pregnant women enrolled in a PCP on the Pacific coast of Colombia, we observed that the opportunity to prevent congenital syphilis was missed in 53.1% of pregnant women because of the lack of maternal screening. We found that the lack of screening through the PCP significantly increased the probability of having newborns with congenital syphilis, and the independent factors associated with not having had >1 screening test through the PCP included characteristics of higher maternal social vulnerability and the late access to the PCP. In addition, we observed that the opportunity to prevent congenital syphilis was missed in 41.5% of pregnant women with syphilis because of the lack of treatment with >1 dose of BPG >30 days before delivery, which increased the probability of having newborns with syphilis.

In the Americas, the prevalence of maternal syphilis and the incidence of congenital syphilis has increased substantially in recent years (*22*,*23*). In our study, we noted a progressive increase in the number of pregnant persons screened for syphilis during 2018–2022. However, the congenital syphilis case-patient ratio and trend could have been influenced by variability in access to the PCP during the years assessed, lack of screening, and potential surveillance biases. For example, screening in women with more risk factors might have increased in 2019, and underreporting also could have occurred, particularly in 2020. Nevertheless, the improvement in screening could indicate progress in syphilis surveillance and control in this area, possibly attributable to the implementation of the EMTCT Plus initiative (*24*) and Colombia’s Resolution 3280 of 2018 (*25*). This resolution established mandatory point-of-care screening using a rapid treponemal test for all pregnant women in each trimester of pregnancy and included administering BPG in case of a positive result (*25*). Although the merits of this screening approach have been debated, implementing point-of-care treponemal tests in low-income settings has been reported to increase the detection and treatment rates of syphilis (*26*). Therefore, that practice could be beneficial in the specific context of the studied region, although additional efforts are still needed to achieve the goal of 95% screening for syphilis in pregnant women. In addition, the defined criteria for congenital syphilis case-patients in Colombia are noteworthy and potentially advantageous (*1*,*9*). That definition could enable patients who might be overlooked using alternative criteria to be identified and treated (*27*). Nevertheless, future studies must be meticulous in evaluating the benefits and risks associated with these approaches, considering the diverse regional contexts (*27*).

Timely prenatal care is crucial for healthy pregnancy outcomes and early syphilis diagnosis (*28*), but certain sociodemographic factors might hinder healthcare professionals from getting to know patients, identifying their vulnerability factors, and providing comprehensive care (*29*,*30*). Independent factors that contributed to the lack of screening included basic or lower level of education, low socioeconomic status, serving as head of household, single marital status, and accessing prenatal care in the second or third trimester of pregnancy. Similar findings on socioeconomic level, prenatal care access, and compliance with screening were reported in the United States (*20*,*31*). In Colombia, late entry into prenatal care was associated with a low socioeconomic stratum (*32*), and a study in China found that single mothers (aOR 1.95) and women who had inadequate prenatal care (aOR 3.61) were at increased risk of having infants with congenital syphilis (*30*).

Several studies have reported that patients’ financial difficulties act as barriers to timely diagnosis of maternal syphilis (*33*,*34*). Those barriers can affect the ability of pregnant women to effectively access healthcare services despite being insured under the public healthcare system (*35*), especially when residing in rural areas within a fragmented healthcare system. Factors such as transportation could play a major role in late access or even lack of access (*18*,*36*), as evidenced in our study, where the population residing in rural areas was very low. Geographic barriers should be assessed in future studies on this region of Colombia.

Socioeconomic status, geographic barriers, low educational level, poor community or family social support, and the specific characteristics of the healthcare system in Colombia can result in limited contact with healthcare personnel and limited information (*37*), which leads to low awareness about the importance of timely prenatal care and detecting diseases such as syphilis early (*11*,*18*,*27*,*33*,*35*–*38*). Therefore, developing healthcare strategies and policies that ensure prompt and effective access to healthcare in the complex context of social determinants of health is imperative (*38*). Those strategies are especially crucial when most of the area’s residents exhibit vulnerable characteristics, and identifying population groups that are most socially vulnerable becomes essential to prioritize effective prevention and control strategies (*31*,*38*–*40*).

On the other hand, we identified factors that did not decrease the probability of receiving >1 maternal syphilis screening, such as a history of >3 pregnancies and Afro-Colombian ethnicity, which is likely related to improvements in healthcare personnel's identification of high-risk pregnancies (*41*,*42*) and the predominance of Afro-Colombian ethnicity in the area. Those findings, combined with progressive improvements in the percentage of screened pregnant persons suggest that previously identified gaps in healthcare providers’ knowledge have gradually been addressed (*11*,*42*).

However, we observed that 41.5% of pregnant women with detected syphilis and with newborns with congenital syphilis did not receive >1 dose of BPG >30 days before delivery. That finding is particularly concerning because in the event of a positive result on the point-of-care treponemal test, administering a dose of BPG immediately is mandatory if the medical history has ruled out BPG allergy (*9*,*25*). A key factor to consider is delayed access to prenatal care, which can lead to late diagnosis and treatment and result in a missed opportunity to prevent congenital syphilis. In addition, although an allergy to penicillin might cause the physician to desensitize the patient to penicillin before initiating treatment (with possible loss of follow-up), true penicillin allergy is extremely rare (*43*), so this allergy is unlikely to result in a missed opportunity to prevent congenital syphilis. Hence, future studies are needed to identify the reasons behind those missed opportunities (*20*).

The missed opportunity to prevent congenital syphilis with BPG administration might be explained in part by documented deficiencies in healthcare professionals’ understanding of the appropriate approach to maternal syphilis (*11*,*42*). Of note, similar missed opportunities have been reported in other settings; a report from the United States found lack of adequate maternal treatment despite timely diagnosis was responsible for 30.7% of missed prevention opportunities (*44*), and in 2022, missed prevention opportunities because of inadequate treatment ranged from 15.7% to 54.5% in various regions of the United States (*20*). Those findings underscore the need for additional research and interventions to ensure the effective treatment of maternal syphilis, given the proven efficacy of BPG (*8*,*27*) and the heightened risk for congenital syphilis when BPG is omitted (*30*). Furthermore, the observation that 58.5% of mothers with diagnosed syphilis and newborns with congenital syphilis had received >1 dose of BPG raises concerns about the adequacy of treatment administered (*9*,*20*,*45*), and potential reinfections (*46*). Consequently, ensuring treatment of sex contacts and tailoring treatment with a penicillin-based regimen initiated >30 days before delivery, with dosing and spacing appropriate for the stage of maternal syphilis, are imperative (*9*,*20*,*45*).

Despite the small sample size and the predominance of participants with characteristics of social vulnerability, this study contributes valuable information to the literature on congenital syphilis. Furthermore, because of the variability in gestational age at entry into the PCP, the results might exhibit bias. However, consistent with other studies, late entry to the PCP was identified as a factor that affected the prevention and control of congenital syphilis (*30*,*31*,*44*). Therefore, we recommend interpreting our findings with caution; future research with larger sample sizes would achieve a more comprehensive understanding of the topic.

Overall, we observed a concerning 53.1% missed opportunity in congenital syphilis prevention because of the lack of maternal screening and a 41.5% missed opportunity because of the lack of maternal treatment. Maternal social vulnerability factors, such as basic or low educational level, low socioeconomic status, being head of the family, single marital status, and late access to prenatal care, increased the probability of not having maternal syphilis screening and having newborns with congenital syphilis. In addition, not having >1 dose of BPG >30 days before delivery, despite being a case-patient with syphilis detected at the PCP, increased the probability of having newborns with syphilis. Therefore, we recommend implementing a comprehensive multidisciplinary approach to identify and address those social vulnerability factors. Furthermore, we suggest intensifying efforts to ensure maternal syphilis is detected in a timely manner and treated adequately to mitigate elevated congenital syphilis incidence on the Pacific coast of Colombia.
